# Risk Factors for Prolonged Mechanical Ventilation and Weaning Failure: A Systematic Review

**DOI:** 10.1159/000525604

**Published:** 2022-08-17

**Authors:** Franziska C. Trudzinski, Benjamin Neetz, Florian Bornitz, Michael Müller, Aline Weis, Dorothea Kronsteiner, Felix J.F. Herth, Noemi Sturm, Vicky Gassmann, Timm Frerk, Claus Neurohr, Alessandro Ghiani, Biljana Joves, Armin Schneider, Joachim Szecsenyi, Selina von Schumann, Jan Meis

**Affiliations:** ^a^Department of Pneumology and Critical Care Medicine, Thoraxklinik, University of Heidelberg, Translational Lung Research Center Heidelberg (TLRC-H), Member of the German Center for Lung Research (DZL), Heidelberg, Germany; ^b^Department of Pneumology and Intensive Care Medicine, Asklepios Klinik Barmbek, Hamburg, Germany; ^c^Department of General Practice and Health Services Research, University Hospital, University of Heidelberg, Heidelberg, Germany; ^d^Institute of Medical Biometry, University of Heidelberg, Heidelberg, Germany; ^e^Institute for Applied Quality Improvement and Research in Health Care GmbH, Göttingen, Germany; ^f^Department of Pulmonology and Respiratory Medicine, Schillerhoehe Lung Clinic (affiliated to the Robert-Bosch-Hospital GmbH, Stuttgart), Gerlingen, Germany; ^g^Pulmonary and Critical Care Medicine Department, Loewenstein Lung Center, Löwenstein, Germany; ^h^Department of Anesthesia and Intensive Care Medicine Waldburg-Zeil Kliniken, Wangen im Allgäu, Germany

**Keywords:** Mechanical ventilation, Weaning, Home mechanical ventilation, Prolonged mechanical ventilation

## Abstract

**Introduction:**

Prolonged mechanical ventilation (PMV) and weaning failure are factors associated with prolonged hospital length of stay and increased morbidity and mortality. In addition to the burden these places on patients and their families, it also imposes high costs on the public health system. The aim of this systematic review was to identify risk factors for PMV and weaning failure.

**Methods:**

The study was conducted according to PRISMA guidelines. After a comprehensive search of the COCHRANE Library, CINHAL, Web of Science, MEDLINE, and the LILACS Database a PubMed request was made on June 8, 2020. Studies that examined risk factors for PMV, defined as mechanical ventilation ≥96 h, weaning failure, and prolonged weaning in German and English were considered eligible; reviews, meta-analyses, and studies in very specific patient populations whose results are not necessarily applicable to the majority of ICU patients as well as pediatric studies were excluded from the analysis. This systematic review was registered in the PROSPERO register under the number CRD42021271038.

**Results:**

Of 532 articles identified, 23 studies with a total of 23,418 patients met the inclusion criteria. Fourteen studies investigated risk factors of PMV including prolonged weaning, 9 studies analyzed risk factors of weaning failure. The concrete definitions of these outcomes varied considerably between studies. For PMV, a variety of risk factors were identified, including comorbidities, site of intubation, various laboratory or blood gas parameters, ventilator settings, functional parameters, and critical care scoring systems. The risk of weaning failure was mainly related to age, previous home mechanical ventilation (HMV), cause of ventilation, and preexisting underlying diseases. Elevated PaCO_2_ values during spontaneous breathing trials were indicative of prolonged weaning and weaning failure.

**Conclusion:**

A direct comparison of risk factors was not possible because of the heterogeneity of the studies. The large number of different definitions and relevant parameters reflects the heterogeneity of patients undergoing PMV and those discharged to HMV after unsuccessful weaning. Multidimensional scores are more likely to reflect the full spectrum of patients ventilated in different ICUs than single risk factors.

## Introduction

Prolonged mechanical ventilation (PMV) and weaning failure are factors associated with prolonged hospital length of stay and increased morbidity and mortality [1]. In addition to the individual challenges that stress patients and their families, the resource-intensive care these patients receive places a significant burden on the public health system [2]. These burdens often persist over the long term, especially in the case of weaning failure with subsequent home mechanical ventilation (HMV).

The Medical Direction of Respiratory Care (NAMDRC) consensus conference defines PMV as at least 21 consecutive days of mechanical ventilation (MV) for six or more hours per day [3]. In the case of difficult weaning from MV, the term “prolonged weaning” is used. Harmonized diagnostic criteria for the weaning process were first established at the International Consensus Conference (ICC) held in April 2005, defining three weaning categories: easy weaning (ICC group 1), difficult weaning (ICC group 2), and prolonged weaning (ICC group 3). According to these criteria, prolonged weaning was defined as the weaning process in patients who have failed at least three weaning attempts or who require more than 7 days of weaning after the first spontaneous breathing trial (SBT) [4]. In 2016, the WIND study group revised these weaning categories and suggested adding an additional “no weaning” category consisting of patients who have never attempted weaning. In addition, the authors recommended that ICC group 3 be divided into group 3a (prolonged weaning with successful weaning seven or more days after the first attempt) and group 3b (prolonged weaning without success) [1]. The current German guideline “Prolonged weaning” further divides patients with weaning failure into a Group 3b, who were discharged with continuous invasive MV and a Group 3c patients who died in hospital [5] Based on these definitions, it can be seen that prolonged weaning is a complex process with outcomes that can range from complete weaning to death.

In specialized weaning centers, successful weaning is possible in about 60% of cases [6, 7]. However, the majority of patients who are discharged to invasive HMV are not presented to such a center beforehand. Therefore, it is important to assess patients' risk of prolonged weaning in order to take specific measures for identified high-risk patients or to plan timely transfer to a specialized weaning facility when needed. We conducted a systematic review to identify risk factors of PMV and weaning failure. The present analysis was carried out as part of the PRiVENT study, which is a prospective multicentric trial with the aim to reduce the number of patients requiring invasive home MV in Baden-Wuerttemberg, Germany.

## Methods

The aim of the study was to identify risk factors for PMV and weaning failure. Study selection was performed following the PRISMA Preferred Reporting Items for Systematic Reviews and Meta-Analyses guidelines [8]. The analysis is registered with the International prospective register of systematic reviews PROSPERO under the number CRD271038.

### Search Strategy

**Fig. 1:**
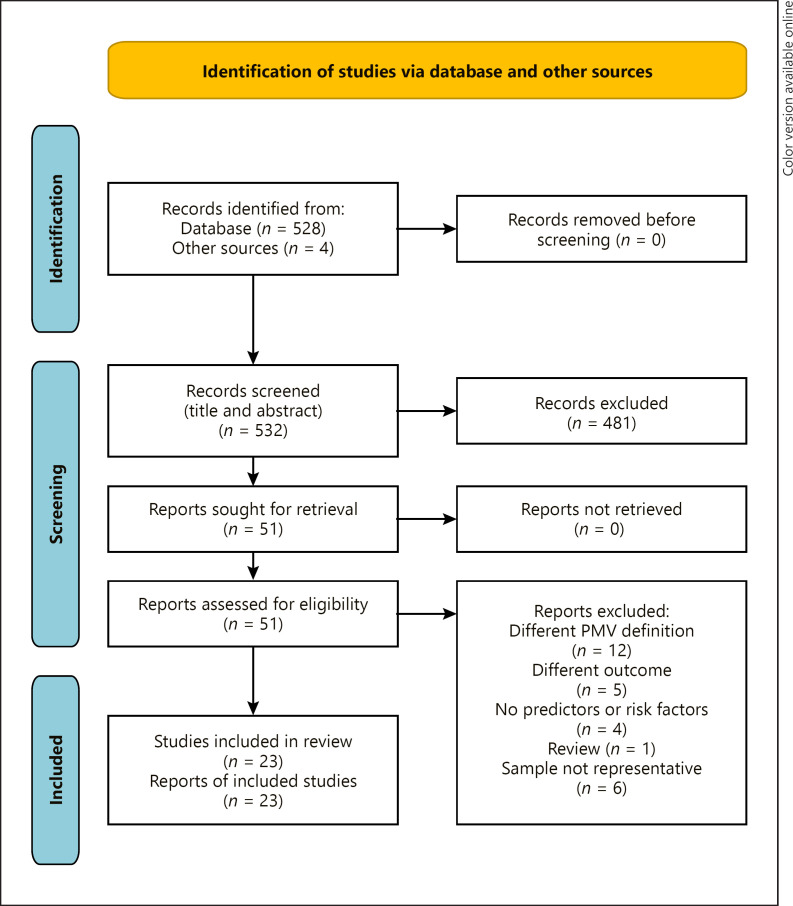
Flowchart of the Preferred Reporting Items for Systematic Reviews and Meta-Analyses. From: [8] http://www.prisma-statement.org/.

In order to meet the methodological standards of systematic reviews and the multidisciplinary requirements of the process of prolonged weaning and weaning failure, the literature search and data extraction were carried out by a team of pulmonologists experienced in respiratory intensive care (F.B., F.C.T.), a respiratory therapist (B.N.), health services researchers (A.W., S.S.), and biostatisticians (D.K., J.M.). In accordance with PRISMA guidelines, a comprehensive search of the COCHRANE Library, CINHAL, Web of Science, MEDLINE, and the LILACS Database was conducted to define the research question and the final PubMed search term (F.B., S.S.). A PubMed enquiry was conducted for (((((((((((PMV) OR (“prolonged mechanical ventilation”)) OR (“long-term mechanical ventilation”)) OR (“prolonged ventilation”)) OR (“ventilator dependence”))) OR (“extend ventilation”)) OR (“prolonged intubation”)) OR (“prolonged acute mechanical ventilation”)) OR ((((((((“difficult weaning”) OR (“inability to wean”)) OR (“prolonged weaning”)) OR (“failure to wean”)) OR (“extend weaning”)) OR (“extubation failure”)) OR (“weaning difficulty”)) OR (“failure to wean”))) AND ((((((risk factors) OR (predict)) OR (“predictive model”)) OR (“logistic regression”)) OR (“risk factors”)) OR (prediction))) AND ((((“intensive care unit”) OR (“critical care”)) OR (“intensive care”)) OR (icu)) on June 8, 2020, start date 2007. The respective PRISMA flowchart is displayed in Figure 1.

### Study Selection Criteria

Studies examining risk factors of PMV, prolonged weaning, and weaning failure in German and English language were considered eligible for inclusion. PMV was defined as MV for at least 96 h. Meta-analyses, systematic reviews, case reports, and case series, studies considering children as well as studies in specific patient populations whose results are not necessarily applicable to the majority of ICU patients, were excluded.

The study selection process consisted of two stages:

1. Title and abstract screening: Two reviewers (F.B., S.S.) used the eligibility criteria to screen titles and abstracts of identified citations for inclusion. In cases where the two reviewers disagreed, a third independent reviewer (B.N.) was consulted to make the final decision.

2. Review of the full texts: the same evaluators (F.B., S.S.) independently reviewed the full texts of the selected publications for their eligibility using the same criteria. The two reviewers then critically discussed their assessments with each other and in cases where no agreement was possible the third reviewer (B.N.) was consulted to make the final decision.

### Data Extraction

Data from eligible studies were extracted in a prepared Microsoft Excel spreadsheet (F.B., B.N., S.S.). The following parameters were systematically assessed: study ID, author, year, title, journal, study design, data basis, inclusion criteria, exclusion criteria, definition of prolonged ventilation, intervention, follow-up period endpoint (definition of “weaning success”), number of patients, statistical methodology, risk factors of PMV or prolonged weaning with respective odds ratios (OR) and 95% confidence intervals, and other endpoints of the studies. Risk factors shown to be predictive in multivariate analyses were given priority when available.

### Quality Assessment

The methodological quality of selected studies and the correctness of extracted data were reviewed by two independent researchers, a biometrician and a clinician (J.M., F.C.T.). Variables that were found to be predictive in multivariate analyses at a significance level of 0.05 by a two-sided test were included in the study. Due to the large heterogeneity in terms of patients included and endpoints studied, we decided not to include odds ratios and respective confidence intervals in the final tables, as they would suggest comparability that is not given. The risk of bias was systematically assessed using an adapted version of the Knowledge Translation Program's prediction worksheet criteria [9].

In accordance with these requirements, three categories were considered.

1. Was a well-defined, representative sample of patients assembled at a common and early point in the course of their disease? Here, the time of intubation was considered a relevant early inclusion time point.

2. Was patient follow-up sufficiently long? Adequate follow-up for the endpoint PMV was defined as 21 days or 3 weaning attempts. For prolonged weaning, a weaning duration of more than 7 days after the first SBT was considered adequate.

3. If subgroups with different prognoses are identified, did adjustment for important prognostic factors take place? Given the large number of factors identified in the various studies, the fact that a multivariate or stratified analysis was performed was already considered positive.

## Results

The initial search yielded 532 studies including 4 additional articles from other sources. Of these, 51 studies were reviewed in full text, and ultimately 23 were included in the final analysis. Of the 28 studies that were not included in the final analyses, 12 were excluded because of different PMV definitions, 5 because of different endpoints, 4 because risk factors were not investigated, and 1 because it was a review article. Six other studies were not included because they examined specific patient populations, such that the results collected were considered unlikely to be generalizable to the majority of ICU patients. The last category concerned studies that exclusively investigated patients with Guillain-Barré syndrome [10, 11], liver [12] and kidney transplant recipients [13], patients who had an implant of a left ventricular assist device [14], and those who had a thoracic trauma with rib fractures [15]. All included studies are listed in Table 1.

### Study Endpoints

Fourteen studies examined risk factors of PMV, including prolonged weaning; 9 examined weaning failure. The definition of PMV was based solely on the duration of ventilation, but the periods reported varied widely between studies. In addition to the most commonly used definition for MV of greater than or equal to 21 days, alternative time periods, such as greater than 7, 14 or greater than 21 days after tracheostomy or 3 SBT and/or a weaning process >7 days were used.

The definitions also differed considerably with regard to weaning failure. Definitions found were failure to wean on day 28, the need to reintubate reconnection to the ventilator within 48 h in case of tracheotomized patients or discharge from the weaning unit with noninvasive or invasive MV. Others assessed weaning failure without further specifications, the death in the course of weaning or the transition to a special unit for HMV initiation. In one case, weaning failure was established by a specialized treatment team including the senior physician. An overview of all definitions used is shown in Table 2.

### Quality Assessment

The quality assessment revealed that intubation, which was considered a relevant time point for early assessment of possible PMV or weaning failure, was not consistently chosen as the time point of inclusion in the studies. Only 5 studies enrolled patients immediately after intubation, and adequate follow-up was also available in 13 of studies.

Three of 23 studies were multicentric, only one of them multinational, and the others monocentric. In all but 3 studies, multivariate analyses were performed with adjustment for relevant confounders.

### Risk Factors for Prolonged MV

Fourteen studies with a total of 8,987 patients investigated risk factors for PMV including prolonged weaning. There were also significant differences in terms of the patient collectives studied. In addition to patients in medical or surgical ICUs without further specification, there were studies that included patients surviving sepsis with respiratory failure, tracheotomized patients undergoing MV, patients admitted to respiratory ICUs, or patients admitted to the ICU after cardiac surgery.

Risk factors identified as relevant for PMV or prolonged weaning include age [1, 16], comorbidities (previous stroke, renal impairment, poor cardiac function, chronic obstructive pulmonary disease [COPD]) [17, 18, 19, 20, 21] and various laboratory parameters (low platelets, elevated blood urea nitrogen, elevated creatinine, low serum albumin, elevated blood glucose levels or hypernatremia) [16, 18, 19, 20, 22, 23]. Blood gas analysis values (lower HCO_3_^-^ or pH values or higher PaCO_2_) as well as parameters of ventilator settings (FiO_2_ ≥ 0.39, level of PEEP) [19, 24] or gas exchange (PaO_2_/FiO_2_ <200 mm Hg) [16] were also identified as relevant. One study prospectively investigated the effects of an intensified insulin therapy (IIT) [22]. In this RCT, the intervention of the IIT reduced the risk of PMV. Some authors developed scoring systems like the I-TRACH score; comprising of 6 variables (intubation in the ICU), tachycardia (heart rate >110), renal dysfunction (blood urea nitrogen >25), acidemia (pH < 7.25), creatinine (>2.0 or >50% increase from baseline values), and decreased HCO_3_ (<20) [18, 20] or the ventilator independence score VIS [23]). The VIS considered Charlson Comorbidity Index (CCI), serum albumin (on hospital admission), g/dL and SOFA score (on ICU admission) [23]. Goligher et al. [25] examined respiratory muscle morphology by serial diaphragmatic ultrasound, thereby, both decreasing and increasing thickness was linked to increased PMV risk [25] The authors suggested that decreasing thickness is associated with abnormally low inspiratory effort and subsequent tissue atrophy, while increasing thickness is associated with excessive effort, possibly related to strain-induced muscle injury [25]. Relevant risk factors for prolonged weaning were baseline Glasgow Coma Scales (GCS) [26], elevated PaCO_2_ at the beginning of [27] and during the first SBT [28], as well as heart rate during the first SBT [28] Hsieh et al. [29] studied 47 different risk factors for prolonged weaning using an artificial neural network. The 47 input features included subject age, gender, scoring systems, such as Acute Physiology and Chronic Health Evaluation II (APACHE-II), Therapeutic Intervention Scoring System (TISS) and GCS, comorbidities, etiology of intubation and respiratory failure, pre-extubation parameters, weaning methods and parameters, and pre-extubation data. Unfortunately, most of their presented results lack proper cross-validation. Table 3 provides an overview of the identified risk factors for PMV.

### Risk Factors for Weaning Failure

Nine studies with a total of 14,431 patients examined risk factors of weaning failure. In addition to the heterogeneous definition of this outcome, the duration of previous ventilation also varied considerably between the studies; in some cases, a previous minimum duration of ventilation was required in others not. Failure of weaning according to the criteria listed in Table 2 occurred in 14.4–51% of cases. The risk factors identified were higher age [9, 30] female gender [31], both low and high BMI values [7, 31], the cause leading to the need for ventilation [7, 32], previous HMV [7], the presence of COPD [7, 9], neuromuscular or thoracic restrictive disease, and arterial hypertension [7] In addition, the Eastern Cooperative Oncology Group performance status was also important [7]. Increased PaCO_2_ values under MV [31] and after the first SBT [6] were also relevant [6, 31]. Table 4 provides an overview of the identified risk factors for weaning failure.

## Discussion

In this systematic review, we examined risk factors for PMV and weaning failure. Of 532 articles identified, 23 studies with a total of 23,418 patients met the inclusion criteria.

The aim of this systematic review was to identify risk factors for PMV and weaning failure. This is an important prerequisite to take specific measures for identified high-risk patients or to plan timely transfer to a specialized weaning facility if needed. One finding of the present work is that the definitions of PMV and prolonged weaning as well as the risk factors examined in the individual studies differ, in part substantially, depending on the patient group studied, which makes simple risk stratification difficult. The majority of studies investigated risk factors for PMV including weaning failure. The definitions of this outcome varied considerably between studies. In addition to the NAMDRC definition, which specifies that PMV starts at day 21 of ventilation [3], several alternative time periods were defined in the studies. On top of this, some authors chose the timing of tracheostomy instead of the start of ventilation as the starting point for counting ventilator time.

Even authors who were aware of the NAMDRC definition often used endpoints other than 21 days for time in their studies. This may be due to the fact that the planning horizon in an intensive care unit is usually much shorter than 21 days, for example when planning a tracheotomy or transfer to a special unit. When analyzing the risk factors for PMV, many of the authors were not concerned with assessing the risk of subsequent home MV, but often with planning further intensive care management or the prognosis in the intensive care unit. A definition that considers duration of ventilation always includes patients who were never considered ready to wean rendering it impossible to distinguish independent risk factors for long-term MV from those that may determine the severity and duration of the underlying disease. Accordingly, the identified risk factors for PMV include factors related to the acute condition, such as critical care scores, acute renal failure, the severity of gas exchange impairment, and low cardiac output Other risk factors included preexisting conditions, such as COPD, previous stroke or the sum of comorbidities determined by the Charlson Comorbidity Index. A notable number of studies came from medical ICUs. In this context, Clark et al. [18, 20] showed that intubation in a medical intensive care unit constitutes an independent risk factor for PMV. These factors suggest a high proportion of comorbidities from the internal medicine field and their contribution to the need for PMV. To address the multitude of relevant parameters, some authors developed specific scores to predict the likelihood of PMV. Multidimensional scores such as the I-TRACH or the Ventilator Independence Score that have been validated specifically for the prediction of PMV show superiority in the risk prediction of this endpoint compared to conventional ICU scores. Compared to others, the VIS score is intended to reflect respiratory muscle strength and endurance. Because this score has only been studied in a monocentric analysis to date, the results need to be verified in further prospective studies. A more technically complex method for determining respiratory muscle morphology is serial diaphragmatic ultrasound, which has been studied by Goligher et al. [25] and has been shown to be informative in predicting PMV risk. The approach is physiologically plausible and practically feasible, but requires some knowledge on the side of the examiner. A sub-analysis of the landmark study by Van den Berghe et al. [33] examined the effects of IIT, targeting blood glucose levels between 80 and 110 mg/dL, on the duration of MV [22]. The authors showed a significant reduction in PMV duration under IIT. However, the results must be regarded as outdated since, in view of the risks of hypoglycemia in critically ill patients, glucose levels between 140 and 180 mg/dL are now recommended [34]. A direct comparison of the studies was not possible because of the large differences in the patient populations studied and the endpoints examined. A comparative analysis with harmonized endpoints would be helpful to identify the most informative parameters.

A special subset of studies on PMV examined prolonged weaning; all of these studies based the diagnosis on the performance of three spontaneous breathing trials and/or a weaning process that lasted more than 7 days as per the ICC criteria [4]. It is therefore conclusive that in two of these studies data were collected during this standardized procedure. Pu et al. [26] identified elevated partial pressures of carbon dioxide at the beginning of the first SBT and Sellares et al. [28] during SBTs as risk factors for prolonged weaning. Another relevant predictor during SBTs was an increased heart rate of more than 105 beats per minute. To account for the multitude of possible variables also related to prolonged weaning, Hsieh et al. [29] developed an artificial neural network that predicts the risk of prolonged weaning based on 47 different risk factors. The approach is interesting, but it remains unclear which of the variables were ultimately decisive. Moreover, such a complex model, which requires the input of a large number of variables, hardly seems feasible in clinical practice.

Prolonged weaning is a complex and often lengthy process. The point at which weaning finally fails is difficult to define. Additionally, there is some disagreement in definitions about whether discharge with noninvasive ventilation (NIV) should be considered as weaning failure. According to the international consensus, both discharges with invasive MV and with NIV are considered manifestations of weaning failure. The authors consider this group of patients as a special category where weaning is still ongoing. As such, they suggest that these patients should not be considered successfully weaned until they are completely weaned from NIV [4]. In the time since then, NIV has developed further. In fact, a relevant number of patients are discharged from hospital with NIV, and the resulting restrictions and costs are significantly lower compared with discharge to home invasive ventilation. These considerations were also reflected in the different definitions of weaning failure used by the 8 included studies considering weaning failure. The definitions for weaning failure ranged from endpoints such as failure to wean at day 28, consistent with previously mentioned definitions of PMV, to an interprofessional diagnostic decision made by a specialized treatment team. However, the mere duration of treatment should not be the only criterion for weaning failure. As many examples revealed, successful weaning is also possible in patients who have been ventilated for an extended period of time prior to weaning in the ICU or transfer to a specialized weaning facility. In addition to older age and poorer general health the determined risk factors of prolonged weaning were mainly factors related to ventilatory failure. Thus, acute worsening or preexisting COPD, thoracic restrictive disease, or neuromuscular disease, as well as previously established NIV, were associated with an unfavorable prognosis. Moreover, elevated PaCO_2_ values after the first SBT or the mechanical power normalized to dynamic compliance of the respiratory system, a surrogate of the work of breathing, prolonged duration of invasive ventilation prior to transfer to a weaning center. The latter factor in particular reflects the need to identify these patients at an early stage.

## Conclusion

With regard to the studies included, the definitions used for PMV as well as weaning failure were very inconsistent and partly overlapping. In addition, the patient populations studied were very heterogeneous. Due to the heterogeneity of the studies, a direct comparison of the studies was impossible. The results reflect the heterogeneity of patients ventilated in different ICUs due to different pathophysiological causes. It is a challenge to harmonize the different definitions of the endpoints to be investigated and at the same time to meet such different needs. The basis for this must be created on the basis of prospective multicenter studies in the interest of the patients concerned. Multidimensional scores covering different aspects of ventilator weaning and intensive care are more likely to reflect the full range of possible risk factors in this heterogeneous patient population than single risk factors.

## Statement of Ethics

An ethics statement is not applicable because this study is based exclusively on published literature.

## Conflict of Interest Statement

Felix J.F. Herth is Editor-in-Chief of “Respiration.” The other authors have no conflicts of interest to declare.

## Funding Sources

The study was carried out as part of the PRiVENT study that is funded by the Federal Joint Committee (Gemeinsamer Bundesausschuss, G-BA) Grant No.01NVF19 023.

## Author Contributions

Franziska C. Trudzinski, screened the full text of identified abstracts for inclusion, reviewed the methodological quality of the selected studies and the correctness of the extracted data. And wrote the first draft of the manuscript, discussed the results and contributed to the final manuscript. Benjamin Neetz, screened the titles and abstracts of identified citations for inclusion, review and editing of the manuscript. Florian Bornitz, developed the project, the main conceptual ideas and the outline, screened the full text of identified abstracts for inclusion, review and editing of the manuscript. Michael Müller, review and editing of the manuscript. Aline Weis, review and editing of the manuscript. Dorothea Kronsteiner, developed the project, the main conceptual ideas and the outline, screened the full text of identified abstracts for inclusion, writing review and editing of the manuscript. Felix J.F. Herth, writing, review and editing of the manuscript. Noemi, Sturm, review and editing of the manuscript. Vicky Gassmann, review and editing of the manuscript. Timm Frerk, review and editing of the manuscript. Claus Neurohr, review and editing of the manuscript. Alessandro Ghiani, review and editing of the manuscript. Biljana Joves, review and editing of the manuscript. Armin Schneider, review and editing of the manuscript. Joachim Szecsenyi, review and editing of the manuscript. Selina von Schumann, developed the project, the main conceptual ideas and the outline, screened the full text of identified abstracts for inclusion, writing review and editing of the manuscript. Jan Meis developed the project, the main conceptual ideas and the outline, reviewed the methodological quality of selected studies and the correctness of extracted data screened the full text of identified abstracts for inclusion, writing review and editing of the manuscript. All authors discussed the results and contributed to the final manuscript.

## Data Availability Statement

All data generated or analyzed during this study are included in this article. Further inquiries can be directed to the corresponding author.

## Figures and Tables

**Table 1:** Overview of identified studies

First author	Year	Study period	Country	Pts., *n*	Endpoint		Quality assesment			
					PMV	weaning failure	study type	sample*	follow-up**	adjustment***
Béduneau et al. [1]	2016	Apr 13 to Jun 13	France, Spain,	2,729	Yes*	_−_	Multicentric, prospective	Yes	Unclear	Yes
			Switzerland							
Chang et al. [19]	2018	Aug 13 to Oct 15	Taiwan	251	Yes	−	Monocentric, retrospective	No	Yes	Yes
Clark et al. [18]	2013	Jan 9 to Jun 10	USA	130	Yes	−	Monocentric, retrospective	Yes	No	Yes
Clark et al. [20]	2018	Unknown	USA	173	Yes	−	Monocentric, prospective	Unclear	No	Yes
Figueroa-Casas et al. [24]										
	2015	Mar 12–14	USA	282	Yes	−	Monocentic, prospective	Yes	No	Yes
Ghiani et al. [31]	2020	Jan 14 to Oct 18	Germany	263	−	Yes	Monocentic, retrospective	No	Yes	Yes
Goligher et al. [25]	2017	May 13 to Jan 16	Canada	191	Yes	−	Monocentic, prospective	Yes	No	Yes
Greenberg et al. [23]	2018	2011–2015	USA	372	Yes	−	Monocentic, retrospective	Unclear	No	Yes
Hermans et al. [22]	2007	Mar 2 to May 05	Belgium	420	Yes	−	Monocentic, prospective sub	No	No	Yes
							Analysis of an RCT [33]			
Hsieh et al. [29]	2019	Dec 9 to Dec 11	Taiwan	3,602	Yes*	−	Monocentic, prospective	Unclear	Yes	Yes
Huang et al. [32]	2019	Jan 12 to Dec. 17	Taiwan	574	−	Yes	Monocentic, retrospective	No	Yes	Yes
Li et al. [9]	2016	Jul 7 to Jul 11	China	302	−	Yes	Multicentric prospective	No	Yes	No
							1-d prevalence study			
Magnet et al. [6]	2018	Jan 9 and Dec 11	Germany	124	−	Yes	Monocentic, retrospective	No	Yes	Yes
Muzaffa et al. [35]	2016	May 14 to Apr 15	India	49	−	Yes	Monocentic, prospective	No	Yes	Yes
Pan et al. [17]	2011	Dec 8 to Nov 9	Taiwan	154	Yes	−	Monocentic, retrospective	Yes	Yes	Yes
Papuzinski et al. [16]	2013	Jun 2011 to Jun 2012	Chile	103	Yes	−	Monocentic, retrospective	Yes	No	Yes
Pu et al. [27]	2015	Jan 12 to Dec 12	China	343	Yes*	−	Multicentric prospective	Unclear	Yes	Yes
Saiphoklang et al. [36]	2018	Jun 12 to Dec 12	Thailand	103	−	Yes	Monocentic, prospective	Unclear	Yes	Yes
Sellares et al. [28]	2010	5-year period	Spain	181	Yes*	−	Monocentic, prospective	Unclear	Yes	Yes
Tseng et al. [37]	2016	Jan 11 to Dec 11	Taiwan	285	−	Yes	Monocentic, prospective	No	Yes	Yes
Wang et al. [21]	2018	Jan 15 − Dec 17	China	56	Yes	−	Monocentic, retrospective	No	No	Yes
Windisch et al. [7]	2020	2011 and 2015	Germany	11,424	−	Yes	Multicentric retrospective	No	Yes	Yes
Wu et al. [38]	2009	Nov 99 to Dec 5	Taiwan	1,307	−	Yes	Monocentic, retrospective	No	Yes	Yes

The risk of bias was systematically assessed using an adapted version of the Knowledge Translation Program's prediction worksheet criteria. In accordance with these requirements, three categories were considered.

* Was a well-defined, representative sample of patients assembled at a common and early point in the course of their disease?

** Was patient follow-up sufficiently long?

*** Given the large number of factors identified in the various studies, the fact that a multivariate or stratified analysis was performed was already considered positive.

**Table 2:** Different definitions for the endpoints

Endpoints	Definitions used
Prolonged MV incl. prolonged weaning	MV >7 days
	MV >7.5 days
	MV >14 days
	MV ≥21 days
	MV ≥21 days after tracheostomy
	3 SBT and/or a weaning process >7 days
	ICC group 2 (difficult weaning) and 3 (prolonged weaning) versus group 1 (simple weaning)

Weaning failure	Death in the course of weaning or the transition to permanent noninvasive or invasive home ventilation
	Transfer to a special unit for HMV initiation
	Failure to wean in 28 days
	Unsuccessful weaning established by a specialized treatment team including a senior physician
	The resumption of ventilatory support within 48 h of discontinuation of MV
	Weaning failure (without further specification)
	The need for reintubation or reconnecting tracheotomized patients to the ventilator within 72 h after weaning from MV, or receiving NIPPV within 72 h after weaning from MV
	Discharge from the weaning unit with invasive MV

MV, mechanical ventilation; SBT, spontaneous breathing trial; ICC, International Consensus Conference; HMV, home mechanical ventilation; NIPPV, Nasal Intermittend Positive Pressure Ventilation.

**Table 3:** Studies identifying risk factors for PMV

First author, ID	Patients	ICU setting	PMV pts. (%)	Risk factors
Béduneau et al. [1]	Pts. receiving MV	36, 10–17-bed. ICUs	399 (22)	Age (year), SOFA at admission, duration of MV before the first separation attempt, medical admissions

Chang et al. [19]	Pts. who survive sepsis/septic shock with respiratory failure	MICU	72 (29)	Previous stroke; platelets ≤150,000/μL, pH ≤ 7.35, FiO_2_ ≥ 0.39 (on admission day 7)

Clark et al. [18]	MICU pts.	16-bed MICU	31 (31.3)	I-TRACH score: intubated in MICU, HR >110/min, BUN >25 mg/dL, pH >7.25, Creatinine >2.0 mg/dL, HCO_3_ >20 mEq/L

Clark et al. [20]	MICU pts.	12-bed MICU	34 (19.7)	I-TRACH score; intubated in MICU, HR >110/min, BUN >25 mg/dL, pH >7.25, creatinine >2.0 mg/dL or a >50% increase from baseline values, HCO_3_ >20 mEq/L

Figueroa-Casas et al. [24]	Medical, surgical, and trauma pts. MV ≥2 consecutive mornings	General ICU	110 (39)	Level of PEEP (from the first 2 days on MV)

Goligher et al. [25]	ICU pts., MV ≥36 h		61 (31.9)	Decrease as well as increase of diaphragm thickness, measured daily by ultrasound

Greenberg et al. [23]	Tracheotomized pts. undergoing MV		103 (44.7)	VIS (ventilator independence score) (on day after, tracheostomy placement), CCI, serum albumin (on hospital admission), g/dL, SOFA score (on ICU admission)

Hermans et al. [22]	Medical and surgical study pts. in the ICU for at least 7 days	MICU	171 (40.7)	Intensive insulin therapy

Hsieh et al. [29]	ICU patients planned for extubation	96 ICU beds; 48 medical, 9 cardiac 39 surgical	311 (8.6)	Artificial neural network model with 47 different 47 clinical risk factors

Pan et al. [17]	Pts. on MV admitted to respiratory ICU	35-bed respiratory ICU	41 (26.6)	Acute kidney injury, MV days before the day of readiness for weaning, RSBI on day of readiness for weaning

Papuzinski et al. [16]	Pts. required invasive MV	General ICU	142 (40.7)	Age, PaO_2_/FiO_2_ >200, Hypernatremia (at intubation), COPD

Pu et al. [26]	Patients intubated, MV ≥24 h, undergoing SBT	Medical and surgical ICUs of 13 hospitals	44 (13)	Glasgow Score PaCO_2_ (at the beginning of the first SBT)

Sellares et al. [28]	Patients intubated, MV ≥48 h, ready to wean	6-bed respiratory ICU	33 (18)	Increased heart rate ≥105 min^−1^, PaCO_2_ ≥54 mm Hg (during the spontaneous breathing trial)

Wang et al. [21]	Pts. who were admitted to the ICU after cardiac surgery		26 (46.4)	Poor cardiac function (ventricular-vascular coupling ratio), lactate at admission

The table shows the studies that examine the risk factors for PMV including prolonged weaning. ICC, International Consensus Conference; ICU, intensive care unit; MICU, medical intensive care unit; MV, mechanical ventilation; FiO_2_, fraction of inspired oxygen; HR, heart rate; BUN, blood urea nitrogen; HCO_3_, bicarbonate; PEEP, positive end-expiratory pressure; VIS, ventilator independence score; CCI, Charlson Comorbidity Index; SAPS II, Simplified Acute Physiology Score; SOFA, Sequential Organ Failure Assessment; SBT, spontaneous breathing trial; PaCO_2_, partial pressure of carbon dioxide; SICU, surgical intensive care Unit; RSBI, rapid shallow breathing index; eGFR, estimated glomerular filtration rate; PaO_2_/FiO_2_, ratio of arterial oxygen partial pressure to fractional inspired oxygen; DPC, Diagnosis Procedure Combination database; RCC, respiratory care center; Acute Physiology and Chronic Health Evaluation.

**Table 4:** Studies identifying risk factors for weaning failure

First author	Patients	ICU setting	Weaning failure Pts. (%)	Risk factors
Ghiani et al. [31]	Pts. with prolonged weaning	Specialized weaning center	137 (52)	Female gender, BMI ≥30 kg/m^2^, mechanical power normalized to dynamic compliance of the respiratory system PaCO_2_ on MV

Huang et al. [32]	Respiratory care center (RCC) pts.	RCC	83 (14.4)	Cause of the respiratory failure that lead to MV

Li et al. [39]	ICU Pts. on MV	55 ICUs	147 (48.7)	Age >74 years, COPD

Magnet et al. [6]	Pts. treated in specialized weaning center receiving MV via tracheostomy		64 (51.6)	PaCO_2_ >45 mm Hg after the first SBT Intubation >30 days Long-term HMV prior to current weaning episode

Muzaffa et al. [35]	Pts. requiring MV ≥21 days, 6 h/day	12-bed medical and surgical ICU	15 (30.6)	Duration of vasopressor support hemodialysis

Saiphoklang et al. [36]	Pts. requiring MV		17 (16.5)	Bronchospasm, pneumonia, malnutrition

Tseng et al. [37]	Non-HF patients admitted to ICU for intensive critical care and ventilator weaning	35-bed MICU	72 (25.2)	Atrial fibrillation on intensive care unit admission

Windisch et al. [7]	Pts. transferred to specialized weaning centers	Specialized weaning centers	2420 (21.2)	Reasons for MV; acute exacerbation of COPD or acute on chronic worsening of neuromuscular disease, older age, low BMI, prolonged duration of invasive ventilation prior to transfer to weaning center, ECOG performance status. Preexisting long-term NIV, COPD, restrictive thoracic disease, NMD, arterial hypertension

Wu et al. [38]	Stable ventilated pts., MV >21 days and failed weaning attempts	24-bed RCC	571 (43.7)	RCC length of stay, APACHE-II, Pimax, Albumin, BUN (within 24 h of admission)

The table shows the studies that examine the risk factors for weaning failure. PaCO_2_, partial pressure of carbon dioxide; RCC, respiratory care center; HF, heart failure; BMI, body mass index; HMV, home mechanical ventilation; MV, mechanical ventilation; SBT, spontaneous breathing trial; ICU, intensive care unit; ECOG, Eastern Cooperative Oncology Group; NMD, neuromuscular disorder; COPD, chronic obstructive pulmonary disease; APACHE-II, Acute Physiology and Chronic Health; Pimax peak inspiratory maximum pressure; BUN, Blood Urea Nitrogen; MICU, MICU, medical intensive care unit.
